# Pediatric Heart Transplant: Initial Experience in a Tertiary Center in Brazil

**DOI:** 10.21470/1678-9741-2021-0483

**Published:** 2022

**Authors:** Francisco Candido Monteiro Cajueiro, Ulisses Alexandre Croti, Alexandra Regina Siscar Barufi, André Luís de Andrade Bodini, Karolyne Barroca Sanches Postigo, Carlos Henrique De Marchi, Fernando Cesar Gimenes Barbosa Santos, Lilian Beani, Bruna Cury Borim, Moacir Fernandes Godoy, Airton Camacho Moscardini

**Affiliations:** 1 Pediatric Cardiology and Cardiovascular Surgery, CardioPedBrasil - Hospital da Criança e Maternidade de São José do Rio Preto, São José do Rio Preto, São Paulo, Brazil.; 2 Pediatric Cardiology and Cardiovascular Surgery, Faculdade de Medicina de São José do Rio Preto, São José do Rio Preto, São Paulo, Brazil.; 3 Pediatric Cardiology, Faculdade de Medicina de São José do Rio Preto, São José do Rio Preto, São Paulo, Brazil.

**Keywords:** Heart Transplantation, Pediatric, Heart Failure, Cardiology, Treatment Outcome

## Abstract

**Introduction:**

Pediatric heart transplantation is the definitive therapy for children with end-stage heart failure. This paper describes our initial experience in pediatric heart transplantation in a tertiary center in Brazil

**Methods:**

This is a historical prospective descriptive cohort study based on a review of the medical records of children undergoing heart transplantation at Hospital de Base and Hospital da Criança e Maternidade de São José do Rio Preto. Variables were displayed as frequency, mean, or median. Statistical analysis and Kaplan-Meier actuarial curve were obtained with the aid of Microsoft® Excel® 2019 and STATSDirect version 3.3.5.

**Results:**

Between January 2010 and December 2020, ten children underwent bicaval orthotopic heart transplantation, 30% of which were under one year of age. Nine patients had end-stage heart failure (International Society for Heart and Lung Transplantation-Heart Failure D) and 50% of the recipients were transplanted under conditions of progressive clinical deterioration (Interagency Registry for Mechanically Assisted Circulatory Support ≤ 2). Forty percent of the recipients had a panel-reactive antibody > 20% on virtual crossmatch. In the postoperative period, 80% of patients required high dose of inotropic support (vasoactive-inotropic score > 10) for > 48 hours. The death-free survival rate at 131 months was 77.1±14.4%. Most patients (88.9%) in late follow-up had an episode of active cytomegalovirus infection. Cellular rejection, with or without clinical repercussion, was present in 44.4% of the patients.

**Conclusion:**

Pediatric heart transplantation produces acceptable and feasible outcomes as definitive therapy for children with end-stage heart failure.

**Table:** 

Abbreviations, Acronyms & Symbols			
BNP	= B-type natriuretic peptide	INTERMACS	= Interagency Registry for Mechanically Assisted Circulatory Support
CMV	= Cytomegalovirus	IQR	= Interquartile range
COVID-19	= Coronavirus disease 2019	ISHLT	= International Society for Heart and Lung Transplantation
CPB	= Cardiopulmonary bypass	IV	= Intravenous
DSA	= Donor-specific antibody	LVEF	= Left ventricular ejection fraction
EBV	= Epstein-Barr virus	MPAP	= Mean pulmonary artery pressure
FAC	= Fractional area change	NO	= Nitric oxide
FiO_2_	= Fraction of inspired oxygen	PCR	= Polymerase chain reaction
HBsAg	= Hepatitis B surface antigen	PEEP	= Positive end-expiratory pressure
HBV	= Hepatitis B virus	PO	= Postoperative
HCV	= Hepatitis C virus	PRA	= Panel-reactive antibody
HIV	= Human immunodeficiency virus	PVR	= Pulmonary vascular resistance
HLA	= Human leukocyte antigen	RV	= Right ventricular
HSV	= Herpes simplex virus	SD	= Standard deviation
HTLV 1-2	= Human T-lymphotropic virus types 1 and 2	TEE	= Transesophageal echocardiography
HTK	= Histidine-tryptophan-ketoglutarate	TPM	= Temporary pacemaker
ICU	= Intensive care unit	VAD	= Ventricular assist device
IgG	= Immunoglobulin G	VDRL	= Venereal disease research laboratory
IgM	= Immunoglobulin M	VIS	= Vasoactive-inotropic score

## INTRODUCTION

Pediatric heart transplantation is the definitive therapy for children with end-stage heart failure^[1]^.

According to the International Society for Heart and Lung Transplantation (ISHLT), by June 2017, more than 14,000 pediatric heart transplants were reported worldwide, of which < 5% were performed in South America^[2]^. Advances in surgical technique, selection criteria, graft preservation, postoperative management, immunosuppression, and the development of ventricular assist devices (VADs) have led to an increase in 10-year post-transplant survival rate to almost 70% in centers of excellence^[3]^.

Between 1990 and 2020, 592 pediatric heart transplants were performed in our country, according to the annual reports of the Associação Brasileira de Transplante de Órgãos. Thirty-nine of those took place in 2020 in 11 transplant centers, mostly located in capitals and large cities^[4]^.

This paper describes our initial experience in pediatric heart transplantation in a Brazilian tertiary center located in a medium-sized city in the State of São Paulo, Brazil.

## METHODS

To describe the clinical characteristics of patients undergoing pediatric heart transplantation at Hospital de Base and Hospital da Criança e Maternidade de São José do Rio Preto (São Paulo, Brazil), a historical prospective descriptive cohort study was carried out, based on a review of the medical records of children undergoing transplantation between January 2010 and December 2020.

This study was approved by our institution’s human research ethics committee via Plataforma Brasil (CAAE: 45710921.5.0000.5415) and term of free and informed consent was waived based on the characteristics of the research (opinion no. 4,713,004 of May 14, 2021).

During the study period, ten children underwent orthotopic heart transplantation, three of which were under one year of age. Their weight ranged between 5.8 and 32 kg. Six were male and none had any genetic syndrome. Perioperative procedures were performed per protocols following the Third Brazilian Guidelines on Heart Transplantation^[4]^.

### Recipients

Transplantation was indicated in patients with end-stage heart failure that were refractory to optimized therapy, with a life expectancy of less than two years and/or unacceptable quality of life. A transdisciplinary approach — medical, physiotherapeutic, nutritional, nursing, and psychosocial — was adopted for a complete assessment of the candidate and clinical management, aiming at preoperative optimization.

All children were primarily diagnosed with cardiomyopathy. No child was transplanted for congenital disease or had undergone previous cardiac surgery. There was a 30% prevalence of failure to thrive with weight-for-height Z-score <-3, categorized as “very low” based on World Health Organization indices.

The pre-transplantation assessment included a thorough review of clinical history, physical examination, chest X-ray, echocardiogram, electrocardiogram, ergospirometry, and hemodynamic evaluation with pulmonary artery catheterization (in three patients). Holter monitoring and an invasive electrophysiology study were performed on a candidate with tachycardiomyopathy.

Patients’ heart failure severity was stratified based on the age-based Modified Ross classification system, Interagency Registry for Mechanically Assisted Circulatory Support (INTERMACS), and ISHLT-Heart Failure for the pediatric population.

Laboratory tests included cultures, cytomegalovirus (CMV) immunoglobulin M (IgM) and immunoglobulin G (IgG) serology, Epstein-Barr virus (EBV) IgM and IgG, toxoplasmosis IgM and IgG, rapid human immunodeficiency virus (HIV) test, anti-hepatitis B surface antigen (HBsAg), anti-hepatitis C virus (HCV), venereal disease research laboratory (VDRL) test, polymerase chain reaction (PCR) for coronavirus disease 2019 (COVID-19) (in the year 2020), panel-reactive antibody (PRA), blood classification, and typing.

Liver (alanine aminotransferase, aspartate transaminase, alkaline phosphatase, gamma-glutamyl transferase, lactate dehydrogenase, albumin-bilirubin score, and prothrombin time) and kidney (urea, creatinine, urinalysis, glomerular filtration rate calculation by Bedside Schwartz Formula, and pediatric Risk, Injury, Failure, Loss, End-stage Renal Disease score) were evaluated, and, if any anomalous phenotypical finding was identified by the pediatric cardiology team, a posterior evaluation by the genetics team was performed; if necessary, a laboratorial investigation by molecular chromosomic tests, including quantitative fluorescent-PCR and single nucleotide polymorphism array, was conducted. Kidney and liver imaging (ultrasound) were performed as needed. Active infection was also ruled out through cultures screening and clinical evaluation. All recipient candidates were screened for HIV, hepatitis A virus, hepatitis B virus (HBV), HCV, EBV, CMV, HTLV 1-2, and since 2020, reverse transcription-PCR testing for COVID-19.

The average waiting time for an organ was 35.5±27.9 days (Table 1).

**Table 1:** Preoperative characteristics of the recipients of pediatric heart transplants performed at Hospital de Base and Hospital da Criança e Maternidade de São José do Rio Preto from 2010 to 2020 (N=10).

Recipients	Frequency, mean (±SD) or median (IQR)
Male (%)	60
Age under one year (%)	30
Weight (kg)	16.3±10.0
Blood group O (%)	70
Cardiomyopathies (%)	100
Time on the transplant list (days)	35.5±27.9
INTERMACS ≤ 2 (%)	50
ISHLT-Heart Failure D (%)	90
Modified Ross Score ≥ 10 (%)	50
LVEF Simpson (%)	16 (12-24)
RV FAC < 35 (%)	40
MPAP > 20 mmHg (%)	50
Received sildenafil (%)	20
Mechanically ventilated (%)	20
Required dialysis (%)	10
PRA > 20% (%)	40
CMV-positive IgG	60
EBV-positive IgG	80

CMV=cytomegalovirus; EBV=Epstein-Barr virus; FAC=fractional area change; IgG=immunoglobulin G; IQR=interquartile range; ISHLT=International Society for Heart and Lung Transplantation; LVEF=left ventricular ejection fraction (calculated using the Simpson method); MPAP=mean pulmonary artery pressure; PRA=panel-reactive antibody; RV=right ventricular; SD=standard deviation

### Donors

Individuals with verified brain death who were determined to be potential organ donors by the Brazilian Central de Notificação, Captação e Distribuição de Órgãos were considered after excluding contraindications that might cause risk to recipients, such as patients with complex heart diseases, evidence of myocardial dysfunction, active bacterial or fungal infections, viral infections, such as HIV, HBV, HCV, and HTLV 1-2, ABO incompatibility, and inadequate graft size for the recipient. The mean age of the donors was 8.9±6.8 years, with 60% comprised of males (Table 2).

**Table 2:** Characteristics of the donors of hearts transplanted at Hospital de Base and Hospital da Criança e Maternidade de São José do Rio Preto from 2010 to 2020 (N=10).

Donors	Frequency, mean (±SD) or median (IQR)
Male (%)	60
Age (years)	8.9±6.8
Weight (kg)	32.2±15.7
Blood group O (%)	80
Death from primary neurological cause (%)	90
Distance from the transplant site (km)	402.5 (276.5-491)

IQR=interquartile range; SD=standard deviation

Donor laboratory evaluation included a virtual crossmatch to detect the presence of donor-specific antibody (DSA), CMV IgM and IgG, toxoplasmosis IgM and IgG, rapid HIV test, anti-HBsAg, anti-HCV, VDRL test, COVID-19 PCR (in 2020), endotracheal, urine, and blood cultures, in addition to strict clinical management to maintain hemodynamically stability in an effort to preserve the viability of the heart.

### Operation

Donors received intravenous (IV) cefuroxime, IV methylprednisolone, and 500 units/kg of heparin prior to aortic clamping, also, Custodiol®-histidine-tryptophan-ketoglutarate (HTK) cardioplegic solution in the aortic root after decompression of the right (partially cutting through the inferior vena cava) and left (opening the right superior pulmonary vein) cavities. To preserve long vascular stumps, cardiectomy was performed by cutting through the vessels (superior and inferior vena cava, aorta, and pulmonary trunk) as distally as possible from the heart. In the case of simultaneous lung collection, the remnants of the left atrium and pulmonary artery were reduced, and the same was applied to the inferior vena cava in case of liver collection. The heart of the donor was kept in a sterile environment composed of a glucophysiological solution at 4°C.

Specimens of the donor’s spleen and lymph nodes were also resected to perform an actual crossmatch.

Recipients received intraoperative antibiotic prophylaxis with IV cefuroxime. Intraoperative monitoring included near-infrared spectroscopy and placement of a transesophageal echocardiography (TEE) probe to be used in the assessment of the graft at any point during the transplant and before (and/or after) weaning from cardiopulmonary bypass (CPB).

All patients underwent median sternotomy, heparinization, and placement of aortic and bicaval CPB cannulas. The perfusate consisted of metabolically corrected and heated Plasmalyte®, and all blood products underwent leukocyte removal and irradiation. CPB was conducted while keeping the patient moderately hypothermic (25 to 28°C). Aortic cross-clamping and explantation of the native heart were performed using the bicaval orthotopic technique. Isoproterenol was routinely initiated for hemodynamic or chronotropic support during weaning and separation from CPB. Milrinone and adrenaline infusions are also used for hemodynamic support as needed intraoperatively. In addition to conventional ultrafiltration, all patients underwent modified ultrafiltration for five to 12 minutes, depending on the patient’s hemodynamic status. Heparin was reversed with protamine (1:1.2), temporary pacemaker wires, chest, and mediastinal tubes were placed, and the chest was closed prior to transporting the patient to the intensive care unit (ICU).

Postoperatively, vasoactive medications such as nitroprussiate, milrinone, epinephrine, norepinephrine, vasopressin, and isoprotherenol were titrated to achieve optimal hemodynamics based on clinical status (*i.e.*, warm, well perfused, urine output, etc.), laboratory evaluation of end-organ function, and oxygen delivery (*i.e.*, arterial blood gas, central venous blood gas and co-oximetry, lactate, blood urea, creatinine, etc.).

Ventilator support and fraction of inspired oxygen (FiO2) were titrated as necessary to achieve normal gas exchange. Mechanical ventilation was guided to optimize metabolic homeostasis and decrease pulmonary vascular resistance (PVR) (FIO2 100%, pH 7.40-45, positive end-expiratory pressure [PEEP] 5 cmH20, tidal volume 6-8 ml/kg, peak inspiratory pressure 10-15 cmH20 above PEEP I/E rate 1:2-3, partial pressure of carbon dioxide [or PCO2] 30-35, partial pressure of oxygen [or PaO2]/FiO2 ratio > 300, and delicate canula fluid aspiration). Nitric oxide (NO) at 20 ppm was initiated intraoperatively if TEE had shown signs of right ventricular (RV) failure or increased PVR. Postoperative pain management and sedation were achieved with titration of opioid, paracetamol, dexmedetomidine, etc.

Patient management decisions were discussed and planned with input from our multidisciplinary team, consisting of nurses, respiratory therapists, infectious disease consultants, immunology consultants, pharmacists, etc., to assure rigorous immunosuppression routine, monitor for signs of rejection in the pediatric population, and prophylaxis against opportunistic infections. All blood derivatives underwent white blood cell removal and irradiation.

Our center’s protocol for timing post-transplant cardiac catheterizations and endomyocardial biopsies is as follows: from zero to four weeks - two biopsies, one at seven and another at 30 days; four to 12 weeks - monthly biopsies; three to 12 months - quarterly biopsies; over 12 months - annual biopsy or when rejection is suspected.

### Immunosuppression

Immunosuppression induction therapy for heart transplant recipients included IV methylprednisolone (20 mg/kg/dose, every 12 hours for 48 hours), cyclosporine (0.1 mg/kg/min, starting one hour before surgery and reinitiated after the end of CPB, subsequently administered orally), and human immunoglobulin (400 mg/kg/dose, every other day, five doses).

Triple oral maintenance therapy included steroids (prednisone 1 mg/kg/dose or prednisolone 1 mg/kg/dose), a calcineurin inhibitor (cyclosporine or tacrolimus at a dose titrated to reach the desired serum levels), and an antiproliferative agent (mycophenolate mofetil 20 to 40 mg/kg/day, 12/12 hours).

Choice of medication regimen for rejection was based on the patients’ clinical status, signs and symptoms (*i.e.*, arrhythmias, fever, and symptoms associated with inadequate cardiac output and oxygen delivery such as nausea, vomiting, dyspnea, etc.), and the underlying etiology, determined by pathological findings of endomyocardial biopsy specimens, (*i.e.*, cellular *vs.* humoral, myocyte inflammation *vs.* graft vasculopathy, and severity based on ISHLT classification), according to ISHLT classification, hemodynamic catheterization, and echo findings (*i.e.*, global dysfunction, decreased cardiac output, new atrioventricular valve regurgitation, increased PVR, etc.).

### Post-Hospital Follow-up

All transplant patients underwent periodic transdisciplinary assessment with outpatient visits and complementary tests including chest X-ray, electrocardiogram, echocardiography, calcineurin inhibitor (cyclosporine or tacrolimus), serology monitoring, and hemodynamic assessment with endomyocardial biopsy. Serum drug monitoring goal for oral cyclosporin was 300-350 ng/mL (0-3 months after transplant), 250-300 ng/mL (3-6 months), 200-300 ng/mL (6-12 months), and 100-200 ng/ml (after one year). Serum drug monitoring goal for tacrolimus was 10-15 ng/ml (0-6 months) and 5-10 ng/ml (after six months).

### Statistics

Nominal qualitative variables were expressed as frequency, and continuous quantitative variables were expressed as mean and standard deviation. Discrete quantitative and continuous quantitative variables without Gaussian distribution were expressed as median and interquartile ranges. Survival analysis was obtained using a Kaplan-Meier actuarial curve. The database was created in Microsoft® Excel® 2019, and the descriptive statistics calculation was performed with the STATSDirect software version 3.3.5 of March 2021.

## RESULTS

**Fig. 1:**
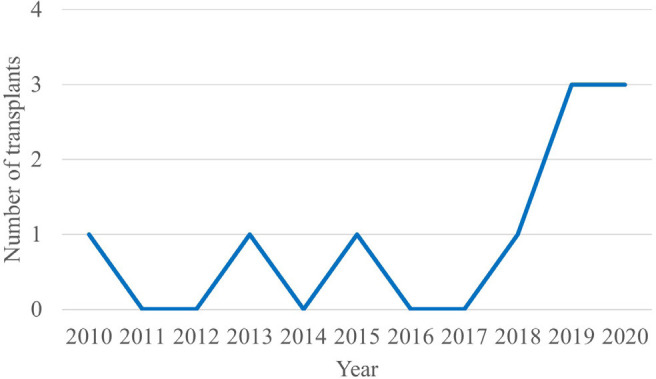
Number of pediatric heart transplants performed at Hospital de Base and Hospital da Criança e Maternidade de São José do Rio Preto from 2010 to 2020.

More than half of the transplants were performed in the last three years, as shown in Figure 1.

Prior to transplant, 50% of the patients waiting for a heart had a modified Ross score ≥ 10 and INTERMACS ≤ 2; 90% of them were in an ICU receiving continuous infusion of inotropic agents for hemodynamic support and were, therefore, assigned to a higher priority level on the waiting list. EBV IgG antibodies were present in 80% of patients, CMV IgG antibodies in 60%, and toxoplasmosis IgG antibodies in 10%.

In the preoperative period, the median left ventricular ejection fraction (LVEF) according to the Simpson’s method was 16 (12-24) %. PRA > 20% was observed in four patients, and none underwent desensitization therapy.

Fifty percent of the recipients had some degree of pulmonary hypertension (mean pulmonary artery pressure [MPAP] > 20 mmHg), and two were transplanted in severe cardiogenic shock, with mechanical ventilatory assistance, dialysis, and high doses of inotropic and vasoactive drugs.

Most of the donors had neurological events that led to brain death. The mean donor/recipient weight ratio was 2.0±0.8 ranging from 0.8 to 3.9. Actual crossmatch after collection did not indicate evidence of DSA.

Table 3 displays the characteristics of the surgical procedure. Donors were at centers located from 276.5-491 km from our center, where the recipients were cared for and would be receiving their heart transplants. The mean CPB time was 101.5±16.5 minutes, and all grafts were transplanted with a total ischemia time of < 240 minutes. Seventy percent of the patients required high-dose vasoactive drugs (vasoactive-inotropic score [VIS] > 10) after weaning from CPB, and two patients required temporary pacing due to inadequate chronotropy in the first six hours. All patients were transfused intraoperatively with packed red blood cells and fresh frozen plasma (even for CPB priming, if indicated).

**Table 3:** Intraoperative data of pediatric heart transplants performed at Hospital de Base and Hospital da Criança e Maternidade de São José do Rio Preto from 2010 to 2020 (N=10).

Intraoperative	Frequency or mean (±SD)
Cold ischemic time (minutes)	118.6±49.1
Warm ischemic time (minutes)	57.0±16.0
Total ischemic time (minutes)	175.6±36.9
CPB time (minutes)	101.5±16.5
TPM required (%)	20
VIS > 10 by the end of surgery (%)	70

CPB=cardiopulmonary bypass; SD=standard deviation; TPM=temporary pacemaker; VIS=vasoactive-inotropic score

* All recipients received isoproterenol (0,05 mcg/kg/min) since cardiopulmonary bypass weaning and have continued for at least 48 postoperative hours.

Eight patients were extubated before the fifth postoperative day, six (60% of the total) in the immediate postoperative period. After the first 48 hours, 80% of transplant recipients maintained VIS > 10. Three patients used NO, and two progressed to renal failure requiring dialysis. Three patients presented intraoperative RV failure with estimated MPAP > 20 mmHg on TEE, therefore NO was added in the operating room until at least 72 hours postoperatively.

Median ICU length of stay was 25.2 (20.2-52.5) days. One child died due multi-organ failure 76 days after surgery (patient 3) secondary to bacteremia and sepsis, further complicated by subsequent fungemia.

In the immediate postoperative period, 40% of the patients had clinical sepsis, with negative blood cultures. No child developed chylothorax, diaphragmatic paralysis, required permanent pacemaker implantation, or underwent unanticipated urgent catheter-based intervention or surgical revision (Table 4).

**Table 4:** Clinical and echocardiographic data from the immediate postoperative period of children undergoing heart transplantation at the Hospital de Base and Hospital da Criança e Maternidade de São José do Rio Preto from 2010 to 2020 (N=10).

Postoperative	Frequency, mean (±SD) or median (IQR)
Mechanical ventilation (days)	1 (1-3.75)
Dialysis required (%)	20
Sepsis (%)	40
Lowest PO LVEF (%)	53.8±11.1
Lowest PO RV FAC (%)	34.7±7.8
Highest PO MPAP (%)	21.3±7.3
Use of NO (%)	30
VIS max > 10 in PO 24 hours (%)	90
VIS max > 10 between PO 24-48 hours (%)	80
ICU length of stay (days)	25.5 (20.2-52.5)
Mortality in ICU (%)	10

FAC=fractional area change; ICU=intensive care unit; IQR=interquartile range; LVEF=left ventricular ejection fraction (calculated using the Simpson method); MPAP=mean pulmonary artery pressure; NO=nitric oxide; PO=postoperative; RV=right ventricular; SD=standard deviation; VIS max=greater vasoactive-inotropic score

Median follow-up time was 15.6 (9.6-24.5) months. During post-hospital follow-up (Table 5), all patients presented some degree of cellular rejection verified by at least one endomyocardial biopsy evidencing ISHLT ≥ 1 and indicating at least mild cellular rejection, though not all patients were symptomatic nor did all of them require significant changes to their immunosuppressive medication. Those that did exhibit significant symptoms and/or require a change, increase, or IV dosing were admitted to the hospital for monitoring during initiation or increased dosing of their immunosuppressive agents.

**Table 5:** Post-hospital follow-up of children who underwent heart transplantation at Hospital de Base and Hospital da Criança e Maternidade de São José do Rio Preto from 2010 to 2020 (N=9).

Post-hospital follow-up	Frequency, mean (±SD) or median (IQR)
Alive at the end of the period reviewed (%)	88.9%
More than one hospitalization per year (%)	44.4%
Sepsis (%)	33.3%
Cellular rejection^1^	100.0%
ISHLT-bio^2^ ≥ 2R (%)	44.4%
BNP3 > 100 (%)	88.9%
Positive IgM titers for CMV*	88.9%
Positive IgM titers for EBV*	11.1%

BNP=B-type natriuretic peptide; CMV=cytomegalovirus; EBV=Epstein-Barr virus; IgM=immunoglobulin M; IQR=interquartile range; ISHLT=International Society for Heart and Lung Transplantation; SD=standard deviation

1 At least one post-hospital endomyocardial biopsy indicating 1R histological pattern.

2 Classification of the ISHLT Biopsy Grading Scale.

3 BNP measured in picograms per milliliter 14 weeks following transplantation.

* Viral activation at some point during post-hospital follow-up.

Non-elective hospitalizations of different etiologies were frequent (on average greater than one hospitalization per year), occurring in 44.4% of patients. The main causes were related to fever, fatigue, decreased oral feeding, respiratory symptoms, and diarrhea, some of which were later identified as early manifestations of CMV.

During late follow-up, eight (88.9%) children had episodes of active CMV infection. None were affected by toxoplasmosis, HIV, HBV, HCV, herpes simplex virus (HSV), or COVID-19. At the end of the study, no child had developed chronic kidney disease, graft vasculopathy, post-transplant lymphoproliferative disease, other malignancies, or underwent re-transplantation.

A second child (patient 2) died 320 days after surgery as a result of humoral rejection.

**Fig. 2:**
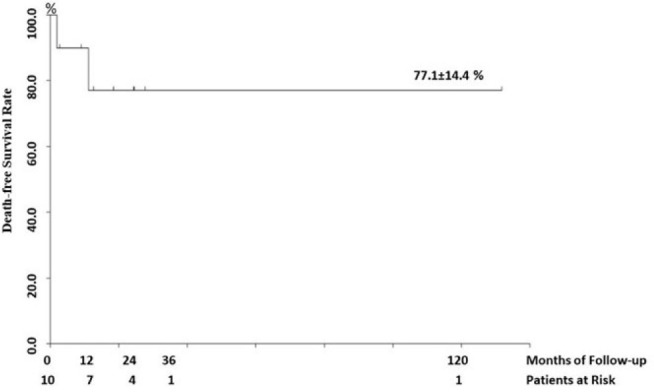
Kaplan-Meier actuarial curve displaying death-free survival rate (until 131 months of follow-up) following pediatric heart transplants performed at Hospital de Base and Hospital da Criança e Maternidade de São José do Rio Preto from 2010 to 2020.

Therefore, overall death-free survival rate was 77.1±14.4% at 131 months of follow-up (Figure 2). Median follow-up time was 15.6 (9.6-24.5) months.

## DISCUSSION

Up until 2015, > 85% of pediatric heart transplants reported to the ISHTL were performed in North America and Europe. Worldwide, most transplant centers are considered low-volume, performing from one to four transplants per year. As in our experience, low-volume centers account for approximately 80% of transplants performed in developing countries^[5]^.

In 2009, the center of Cardiologia e Cirurgia Cardiovascular Pediátrica of the Hospital de Base and Hospital da Criança e Maternidade de São José do Rio Preto initiated a scientific-educational partnership with the American non-profit organization Children’s HeartLink (or CHL), whose mission is to foster the development of pediatric cardiology care in developing countries. This alliance allowed the center to be later included in the International Quality Improvement Collaborative for Congenital Heart Disease (or IQIC), an initiative whose purpose is to identify the contributing factors to postoperative child mortality following cardiovascular surgery^[6]^.

Over time, the elimination of weaknesses and the institutional improvement led to an increase in the number and complexity of surgeries, in addition to a significant decrease in infection and mortality rates. This increase in quality is reiterated by the increase in the number of transplants performed in the last three years.

Age stratification of recipients shows that, in our sample, 30% of transplant recipients were younger than one year of age, and 40% were between one and ten years of age, a ratio compatible with medium (5-9) and large (10+) annual volume centers around the planet^[7]^.

There are geographical differences regarding age and primary diagnosis of recipients. In North America, the majority of pediatric heart transplant recipients are infants and children with congenital heart disease, whereas in Europe and other parts of the world, dilated cardiomyopathy accounts for the highest proportion^[8]^. In our study, the predominance of recipients transplanted due to cardiomyopathies is in line with data from developing countries, like ours. In the 2000s, in São Paulo, Azeka et al.^[9]^ reported that cardiomyopathies had been the indication for heart transplant in 97.3% of children over one year of age.

In the pediatric primary care setting, recognizing and diagnosing cardiomyopathy can be quite challenging, especially since most of those patients have structurally normal hearts and are not followed by a pediatric cardiologist. Reports of multiple hospitalizations for the treatment of nonspecific respiratory, digestive, and infectious conditions are frequent. With the delay in being referred to a tertiary center, 90% of the study patients were hospitalized in end-stage heart failure (ISHLT-Heart Failure D). By the time of transplant, 50% of recipients had demonstrated significant clinical deterioration despite IV inotropic therapy (INTERMACS 2), some of which developed refractory, uncompensated cardiogenic shock (INTERMACS 1), situations where a short-term VAD would be indicated as a bridge for decision in selected cases^[10]^.

In countries with limited resources, providing advanced care in pediatric cardiology and cardiac surgery is a challenge^[11]^. During the study, the center did not have circulatory assist devices to provide metabolic rescue to severe patients, which, in some cases, was a temporary contraindication to the listing and, in others, required the transplantation to be carried out before the clinical condition became irreversible.

In a similar situation, Jatene et al.^[12]^ described the evolution of 22 children with dilated cardiomyopathy who presented uncompensated cardiogenic shock while waiting for transplantation; 42.9% of these children died while on the waiting list and eight transplants were performed. The clinical course of those patients who underwent orthotopic heart transplant emergently was similar to those that underwent transplant electively.

Across the world, the number of children in need of heart transplants exceeds the number of donors, and mortality on the waiting list remains high. It is important to be able to identify which recipients should be prioritized on the waiting list before their condition becomes too severe to survive a transplant (or even until an organ becomes available) and from which donors in borderline clinical condition, the so-called marginal donors, it is justifiable to leverage the risk of accepting a suboptimal organ^[13]^.

In 2020, the ISHLT published a consensus focusing on donor management and organ acceptability, identifying features that could negatively impact short and long-term outcomes. Among such features, the following must be highlighted: donor more than five years older than the chronological age of an adolescent recipient or donor > 25 years old, donor LVEF < 40%, West Nile virus infection, Chagas disease (*Trypanosoma cruzi*), untreated fungal infections, non-bacterial meningoencephalitis, hematologic malignancy, and ischemic time > 4 hours. Interestingly various donor factors are not associated (or literature is still inconclusive) with negative outcomes: donor/recipient weight ratio between 0.6 and 3.0; bacterial infections, donors killed by the *Influenzae* virus, diabetes, hypertension, history of anaphylaxis, other tumors, genetic syndromes, history of cardiopulmonary resuscitation, cause of death, use of inotropic agents and vasopressors, electrocardiographic alterations, and troponin, creatine kinase myocardial band, B-type natriuretic peptide (BNP), and pro-BNP levels^[14]^.

In our casuistic, we identified one transplantation performed with a donor-recipient age difference greater than five years without negative consequences observed during the follow-up period.

When analyzing data from the Pediatric Heart Transplant Study (or PHTS) registry, Das et al.^[15]^ identified that sensitization of receptors to human leukocyte antigen (HLA) is common and about 26% had a PRA > 20% for HLA class I or II before transplantation. Patients with PRA > 20% were more likely to have congenital heart disease, to have undergone previous cardiac surgery, requiring extracorporeal membrane oxygenation support at the time they were listed for transplant (or at any point thereafter, until the time of transplant), and to have waited longer for an organ than those with PRA < 20%. PRA > 50% was independently associated with shorter graft survival during the first month following transplantation, with greater probability of a positive crossmatch, and with the presence of DSA. Forty percent of patients in our sample had PRA > 20%, and there were no patients with specific antibodies against the donor.

Long-term graft survival and recipient survival are also dependent on graft preservation strategies. The use of a hypothermic HTK solution applied for a certain period and at low pressure to the aortic root has been the center’s standard since the first transplantation. In 2020, Shaw et al.^[16]^ compared the 30-day, one-year, and long-term survival (10 years after heart transplantation) of 3,102 children undergoing heart transplantation whose grafts had been preserved with different solutions. The authors found no significant differences between the following solutions: Celsior, University of Wisconsin, and Custodiol®-HTK.

Approximately 40% of pediatric heart transplant recipients have arrhythmias. Those who develop bradyarrhythmia may need temporary pacing, if they do not have an adequate chronotropic response to isoproterenol in the immediate postoperative period, or permanent pacemaker implantation, if persistent. In a meta-analysis of 7,198 pediatric heart transplant recipients, Mylonas et al.^[17]^ reported a prevalence of 1.9% (137) of that requiring permanent pacemaker implantation. Sinus node dysfunction and complete atrioventricular block were the main indications, more frequently in patients operated using the Lower-Shumway technique. We used the bicaval technique in all transplant recipients and two children (20%) that required a temporary pacemaker for a few hours in the immediate postoperative period. No patient required placement of a permanent pacemaker.

Acute RV dysfunction is a potential complication after heart transplant. It may occur since intraoperative graft reperfusion and encompass/covers a broad spectrum of presentation and can be triggered primarily or consequently to left ventricular failure. Mild RV dysfunction frequently occurs in the early postoperative period (managed by ventilation, hemodynamical optimization, and pharmacological measures) but, in worst cases, severe RV dysfunction can culminate with graft failure requiring VAD or even waitlist relocation for a new organ. Factors such as prolonged graft ischemia time, prolonged cardiopulmonary resuscitation of the donor, mechanical ventilation, and pre-transplant elevated PVR are risk-increasing for fatal outcomes^[18]^.

Forty percent of our recipients had an RV fractional area change (FAC) < 35%, and 50% had MPAP > 20 mmHg as their worst echocardiographic measurements while in the ICU. Association with NO was required in three of them. In 2019, a study involving 2,833 patients in 28 American centers described a frequency of 36.5% NO use after transplantation. Prolonged use (> 5 days) was associated with decreased survival and a significant increase in costs and was considered a marker of severity^[19]^. It is important to highlight that acute RV dysfunction is a physiopathologic condition distinct from hyperacute humoral rejection, a rare (nowadays), dramatic, and often lethal immune-mediated response usually occurring 24 hours post-transplantation.

As for pharmacological support in the first 24 hours after transplantation, 90% of the children required high doses of inotropic and vasoactive drugs (VIS > 10), and at 48 postoperative hours, 80% of all patients remained at VIS > 10. In 2019, Tadros et al.^[20]^ reported that patients with a high maximum and medium VIS in the first 24 hours and 24-48 hours had longer hospital length of stays, prolonged need for mechanical ventilation, and inotropic support. They also tended to have more adverse cardiac events and were more likely to have suffered acute kidney injury. The cutoff point for risk stratification based on a VIS of 10 is suitable for pediatric cardiac recipients (area under the receiver operating characteristic curve > 0.8).

The greatest risk of viral infections happens around six to eight weeks after surgery. Eight out of nine patients (88.9%) discharged from the hospital had episodes of active CMV infection, requiring specific antiviral therapy. CMV infection is frequent in the pediatric population, however, death resulting from this specific cause is uncommon. Seronegative recipients receiving organs from seropositive donors are at greater risk. Other opportunistic viral agents include HSV, varicella-zoster virus, influenza viruses, and EBV^[21]^.

Four of the nine patients (44.4%) in post-hospital follow-up underwent endomyocardial biopsies (elective or urgent), indicating a histological pattern of moderate or severe cell rejection. These patients were readmitted for adjustment of immunosuppression or initiation of rejection therapy based on other clinical and echocardiographic findings with corroborating findings on physical exam. According to the ISHLT, > 10% of transplant patients experience episodes of acute rejection within the first year requiring treatment and/or hospitalization^[22]^.

The death-free survival rate was 77.1±14.4% at 131 months of follow-up. The standard error is of probability (survival rate), not of the absolute number of survivors. The main causes of death in children following heart transplantation were graft dysfunction, infections (non-CMV), acute rejection, and graft vasculopathy. Their frequency varies according to the time elapsed after transplant. Infections (non-CMV) and acute graft rejection rank second and third among the leading causes of death between 31 days and one year following heart transplantation^[23]^.

Two of our patients died following transplant, with etiology, clinical course, and cause of death similar to what has been described by others in the literature.

The first patient was a 12-year-old child; it was an incredibly high-risk transplant due to extremely poor pre-transplant clinical and nutritional statuses (Body Mass Index Z-Score-2.1, idiopathic dilated cardiomyopathy, INTERMACS 1, LVEF 10%, FAC > 35%, MPAP 30 mmHg, presenting hepatic disfunction, and requiring vasoactive infusions, mechanical ventilation, and dialysis). Her waitlist time was of ten days, the female donor was 15 years old, weighting 55 kg, who died of hemorrhagic stroke, with total ischemia time of 235 minutes (cold, 190 minutes; warm, 45 minutes) and VIS 32 at recipient ICU admission. Cause of death was sepsis-associated multi-organ failure at 76 days post-transplant. This patient presented bacteremia with positive urine and blood cultures for *Pseudomonas aeruginosa* and went on to develop fungemia (non-*albicans Candida* sp.). During ICU course, there were multiple invasive devices including indwelling central lines, nasoenteral feeding tube, total parenteral nutrition, orotraqueal tube, tracheostomy, peritoneal dialysis catheter, Foley catheter, and multiple thoracic drainages, among other procedures. Multiple efforts had been made to optimize hemodynamic and infectious scenarios. During the studied period, our center did not have circulatory assist devices to provide metabolic and hemodynamic rescue to severely ill patients.

The second patient was a eutrophic toddler transplanted at 22 months of age, diagnosed with idiopathic dilated cardiomyopathy, INTERMACS 3, LVEF 11%, FAC 35%, MPAP 14 mmHg, using minimal vasoactive support. He had a waitlist time of 12 days, a female donor of seven years old, weighting 26 kg, and he was extubated on the third postoperative day and submitted to standard immunosuppression protocol with hospital discharge without major complications. After hospital discharge, there were five non-programed readmissions related to CMV infection/diagnostic/control. He died 356 days post-transplant, likely a consequence of noncompliance with the immunosuppressant medication regimen by the patient’s guardians. The patient had evidence of significant humoral rejection, as immunohistochemical staining of endomyocardial biopsy specimens were positive for C4d on myocardial microvasculature.

Successful intermediate and long-term pediatric heart transplant is highly dependent on patients’ parents/caregivers being vigilant with the child’s immunosuppression regimen and attending follow-up cardiology appointments after discharge from the hospital. Stone et al.^[24]^ observed that children cared for by parents with an unfavorable psychosocial evaluation had a 2.4 times greater risk of experiencing episodes of acute rejection and 2.9 times greater risk of having subtherapeutic levels of calcineurin inhibitor (cyclosporine or tacrolimus). These at-risk children could benefit from in-home monitoring and solid psychosocial support. Considering the literature and our clinical experience with late death, we have become more rigorous in our evaluation of patients’ psychosocial and economic situation prior to offering heart transplantation as a treatment option for our patients with cardiomyopathy and end-stage heart failure.

### Limitations

The purpose of this study was to describe our initial experience with heart transplantation for pediatric patients with cardiomyopathy and end-stage heart failure. Due to the small number of patients, the study is not adequately powered to draw any conclusions with respect to possible and statistically significant associations between variables. Further, data was extracted from review of medical records, some of which were older and difficult to obtain. Another limitation of our study is that the VIS calculation does not include isoproterenol, which is essential to the perioperative medical management of pediatric heart transplant recipients. Therefore, VIS does not reflect all of the vasoactive medication required by any given patient.

Finally, most of the transplants took place in the last three years, therefore the sample follow-up time is short. This explains the lack of late complications, such as chronic kidney disease, graft vasculopathy, post-transplant lymphoproliferative disease, other malignancies, or patients undergoing re-transplantation.

## CONCLUSION

Pediatric heart transplantation is feasible and is consistently associated with excellent immediate, short-, and medium-term outcomes when employed as definitive therapy for children with cardiomyopathy and end-stage heart failure. It is very important to consider the risk based on the patient’s clinical and nutritional statuses. Further, success is dependent on compliance with immunosuppressive medication regimens and attendance at scheduled follow-up appointments.

**Table:** 

Authors’Roles & Responsibilities	
FCMC	Substantial contributions to the conception or design of the work; or the acquisition, analysis, or interpretation of data for the work; drafting the work or revising it critically for important intellectual content; agreement to be accountable for all aspects of the work in ensuring that questions related to the accuracy or integrity of any part of the work are appropriately investigated and resolved; final approval of the version to be published
UAC	Substantial contributions to the conception or design of the work; or the acquisition, analysis, or interpretation of data for the work; drafting the work or revising it critically for important intellectual content; agreement to be accountable for all aspects of the work in ensuring that questions related to the accuracy or integrity of any part of the work are appropriately investigated and resolved; final approval of the version to be published
ARSB	Substantial contributions to the conception or design of the work; or the acquisition, analysis, or interpretation of data for the work; drafting the work or revising it critically for important intellectual content; agreement to be accountable for all aspects of the work in ensuring that questions related to the accuracy or integrity of any part of the work are appropriately investigated and resolved; final approval of the version to be published
ALAB	Substantial contributions to the conception or design of the work; or the acquisition, analysis, or interpretation of data for the work; drafting the work or revising it critically for important intellectual content; agreement to be accountable for all aspects of the work in ensuring that questions related to the accuracy or integrity of any part of the work are appropriately investigated and resolved; final approval of the version to be published
KBSP	Substantial contributions to the conception or design of the work; or the acquisition, analysis, or interpretation of data for the work; drafting the work or revising it critically for important intellectual content; agreement to be accountable for all aspects of the work in ensuring that questions related to the accuracy or integrity of any part of the work are appropriately investigated and resolved; final approval of the version to be published
CHDM	Substantial contributions to the conception or design of the work; or the acquisition, analysis, or interpretation of data for the work; drafting the work or revising it critically for important intellectual content; agreement to be accountable for all aspects of the work in ensuring that questions related to the accuracy or integrity of any part of the work are appropriately investigated and resolved; final approval of the version to be published
FCGBS	Substantial contributions to the conception or design of the work; or the acquisition, analysis, or interpretation of data for the work; drafting the work or revising it critically for important intellectual content; agreement to be accountable for all aspects of the work in ensuring that questions related to the accuracy or integrity of any part of the work are appropriately investigated and resolved; final approval of the version to be published
LB	Substantial contributions to the conception or design of the work; or the acquisition, analysis, or interpretation of data for the work; drafting the work or revising it critically for important intellectual content; agreement to be accountable for all aspects of the work in ensuring that questions related to the accuracy or integrity of any part of the work are appropriately investigated and resolved; final approval of the version to be published
BCB	Substantial contributions to the conception or design of the work; or the acquisition, analysis, or interpretation of data for the work; drafting the work or revising it critically for important intellectual content; agreement to be accountable for all aspects of the work in ensuring that questions related to the accuracy or integrity of any part of the work are appropriately investigated and resolved; final approval of the version to be published
MFG	Substantial contributions to the conception or design of the work; or the acquisition, analysis, or interpretation of data for the work; drafting the work or revising it critically for important intellectual content; agreement to be accountable for all aspects of the work in ensuring that questions related to the accuracy or integrity of any part of the work are appropriately investigated and resolved; final approval of the version to be published
ACM	Substantial contributions to the conception or design of the work; or the acquisition, analysis, or interpretation of data for the work; drafting the work or revising it critically for important intellectual content; agreement to be accountable for all aspects of the work in ensuring that questions related to the accuracy or integrity of any part of the work are appropriately investigated and resolved; final approval of the version to be published
